# Activity levels of female *Triatoma infestans* change depending on physiological condition

**DOI:** 10.1186/s13071-018-3117-9

**Published:** 2018-10-01

**Authors:** Luciana Abrahan, Pablo Lopez, Ivana Amelotti, María José Cavallo, Raúl Stariolo, Silvia Catalá, Gerardo Cueto, Verónica Valentinuzzi

**Affiliations:** 1 Centro Regional de Investigaciones Científicas y Transferencia Tecnológica de La Rioja (CRILAR), UNLAR, SEGEMAR, UNCa, CONICET, Anillaco, La Rioja, Argentina; 2 Universidad Nacional de La Rioja, La Rioja, Argentina; 3 Centro de Referencia de Vectores, Santa María de Punilla, Córdoba, Argentina; 4 Instituto de Ecología, Genética y Evolución de Buenos Aires, Buenos Aires, Argentina

**Keywords:** Triatomines, Kissing bugs, Chagas disease, Active dispersal

## Abstract

**Background:**

*Triatoma infestans* (Hemiptera: Reduviidae) is the main vector of *Trypanosoma cruzi*, the etiological agent of Chagas disease, in South America. Active dispersal of this vector is the principal cause of recolonization of human dwellings previously treated with insecticides. Due to the persistence of vector populations and their movement between habitats, dispersive behavior studies are important for understanding the epidemiology of Chagas disease. The aim of this study was to analyze the relationship among *T. infestans* females’ activity levels according to their physiological conditions.

**Results:**

Two groups of insects were used, unfed and fed females. Each was composed of three subgroups in relation to the reproductive state: fifth-stage nymphs, virgin and fertilized females. There was a significant interaction between reproductive and nutritional states among *T. infestans* female’ activity levels. During the experiments, unfed and fed nymphs remained inactive. Virgin females showed a dual behavior in their movement; fasted insects were more active. Fertilized females, both fed and unfed, were always active.

**Conclusion:**

The reproductive and nutritional conditions of *T. infestans* females affect their activity levels. When females with different reproductive states remained together, fertilized females showed permanent activity levels, suggesting that this subgroup of females represents the highest epidemiological risk as colonizers of human dwellings.

**Electronic supplementary material:**

The online version of this article (10.1186/s13071-018-3117-9) contains supplementary material, which is available to authorized users.

## Background

Active dispersal is a key process in ecology that affects the dynamics and persistence of populations. Evolutionary models and empirical studies, aimed at elucidating the causes of animal dispersal, have shown that various factors, such as intraspecific competition, inbreeding risk, food availability, population density, sex ratio and habitat isolation, may encourage dispersal activity [1, 2]. In triatomines, active dispersal is of great importance for the vectorial control of Chagas disease [3]. In triatomines with an affinity to the human habitat, active dispersal may lead to the recolonization of ecotypes previously treated with insecticide [3–6]. Within triatomines, *Triatoma infestans* is the main vector of *Trypanosoma cruzi*, the etiological agent of Chagas disease, in the Southern Cone of South America. In this species, dispersing females are able to recolonize habitats, representing a high risk as reinfestants of human dwellings [7]. Consequently, knowledge of *T. infestans* females’ behavior is of paramount epidemiological importance.

In *T. infestans*, active dispersal may occur by flight [7–9] or walking [7]. Active dispersal has been mainly associated with the need to reproduce and the search for food [3]. Many reports have suggested that dispersive behavior varies between sexes. Some authors reported higher flight dispersal levels in females [10–14], while others reported that flying males dispersed more [7–9, 15, 16]. This is in accordance with the fact that males have an active reproductive conduct generated by females’ metasternal gland odors, while females seem to limit their reproductive behavior in accepting or rejecting male mating attempts [17–21].

The dispersal of triatomines by walking has been less studied. For *T. infestans*, Abrahan et al. [7] reported that males disperse by flying, while females tended to walk. Furthermore, within females, fertilized ones showed a predisposition to disperse more than virgins and fifth nymphs [7]. Nutritional requirements could be one of the factors that determine these different dispersal tendencies. Nymphs usually have to feed more than once to be able to molt to the next stage [22–24], while adult females require nutrients to produce eggs [25]. That is, fasting conditions could yield an extension of nymphal stages (mainly fifth stage), and a reduction in fecundity: conditions that increase dispersal probabilities resulting in a population density regulation strategy [3]. Understanding these factors that induce female dispersal allows further comprehension of their colonization role.

Females have a higher colonizing efficiency than males, and this efficiency varies with their reproductive and nutritional states. When fertilized, females can start a new colony, while virgins can successfully colonize only if they find a male in the new habitat. Field studies showed that 88% of walking females were fertilized, carrying numerous eggs within the oviducts, and/or with blood reserves in the midgut [7]. The lack of control of the diverse factors that affect field dispersal, determine the need to evaluate this behavior in controlled laboratory conditions. Since *T. infestans* is a gregarious species in the field, it is common to find the five nymphal stages and adults within the same restricted area and sharing the same refuge [26]. These reasons led us to query whether fertilized females, maintained in laboratory conditions, express different behavior when other females in different reproductive states are together. Additionally, host presence or absence is a crucial environmental factor affecting dispersal [27, 28]. The response of insects to the presence of a potential host depends on the multimodal integration of a variety of external cues, insect physiological states and individual previous experience [29].

Our purpose was to determine if there was a relationship among *T. infestans* females’ activity levels, according to their nutritional and reproductive states, in the absence and in the presence of a host. For this study, activity level was considered an indicator of the potential for dispersal, generated by the motivation of the females to exit their rest refuge.

## Methods

### Insects

*Triatoma infestans* specimens were F1 descendants of insects collected in the locality of Chuña, Córdoba, supplied by the Centro de Referencia de Vectores (CeReVe) from the National Health Ministry of Argentina (Córdoba, Argentina). Insects were kept under controlled temperature (27 ± 2 °C) and humidity conditions (45 ± 5%), in a 12:12 h light-dark cycle, and fed weekly on hens. Nymph sex was determined by the last abdominal segments [30]. Virgin females were maintained without contact with males. In order to obtain fertilized females, virgin females were placed with males for 2-day intervals; the presence of the spermatophore was the mating sign.

Two groups with different nutritional states were used: unfed females (after fasting for two weeks) and fed females (fed 48 h before the experiment). Each group was placed in a different arena. There were 3 replicates for the unfed group and 2 replicates for the fed group. Each of these groups was composed of 3 subgroups that varied in their reproductive state: 10 fertilized females, 10 virgin females and 10 fifth-stage nymph females. In the latter, the morphophysiological characteristics (absence of a scutellum, wings and mature sex organs) allowed us to consider this subgroup as a sexually immature version of the adult females. Each subgroup was marked with white acrylic paint in different parts of their dorsal surface to allow identification when released together in the arena. Insects were used once and discarded afterwards.

### Experimental design

**Fig. 1:**
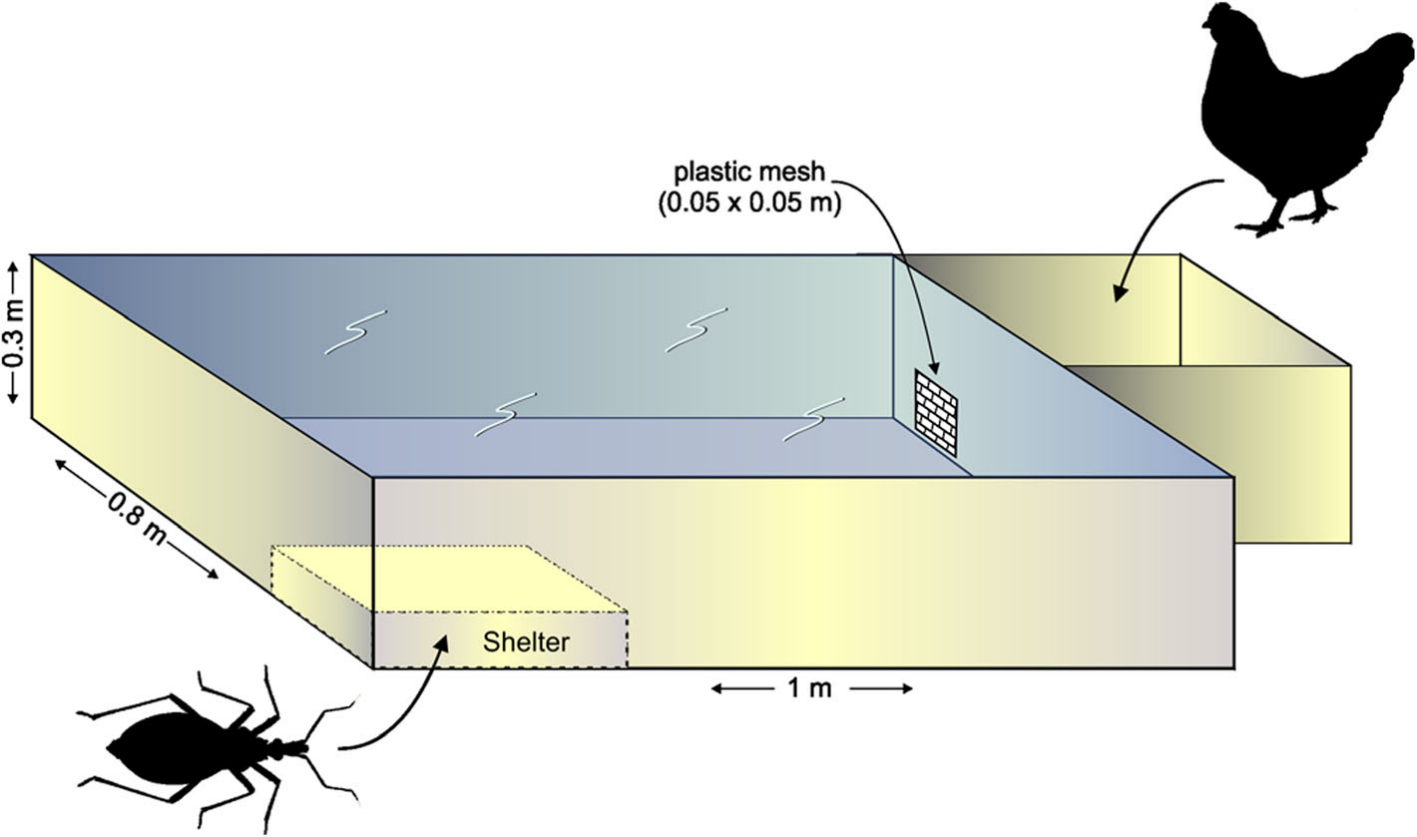
Schematic representation of the experimental arena

Wooden behavioral arenas (80 × 100 × 30 cm, Fig. 1) were used, similar to those reported by Lorenzo & Lazzari [31]. A meshed covered top prevented escape. Outside and contiguous to one of the shorter sides of the rectangle-like arena, close to a corner, was a cube-like compartment (25 × 25 × 30 cm) in which a hen could be housed according to the experimental phase. This hen compartment was connected to the arena through an opening, covered with a plastic mesh. A shelter was placed as far as possible from the hen compartment, in the diagonally opposite corner. The shelter consisted of a 10 × 15 × 3 cm cardboard box with a piece of corrugated paper inside.

At 80 cm above the arena, a high-definition video camera and a specific illumination system were mounted. The latter consisted of 75 LEDs emitting at 660 nm, resulting in a light intensity of 1.9 μW/cm^2^. Preliminary studies (Abrahan et al., unpublished data) showed that this wavelength did not generate photonegative behavior, using a similar experimental setup as in [32]. The light intensity used, measured by radiometer (Skye Instruments®, Llandrindod Wells, Powys, UK), allowed good recording and visualization of the individuals. The video system was connected to a computer that continuously recorded insect behavior in each experiment. All equipment was located in a room at constant temperature (27 ± 3 °C), relative humidity (40–50%) and with a light-dark cycle of 12:12 h (lights on at 19:00 h).

At the beginning of each trial, insects were placed together inside the shelter of the arena from which they could exit and enter freely. For each nutritional group, in each replicate, 30 females (10 of each subgroup) remained in the arena for 38 h (two nights and one day of monitoring). During the first night, the host was absent. During the second night, a hen was housed in the corresponding compartment so that the triatomines could perceive the odors, and the heat produced by the bird [33]. This host species was used because hens are considered one of the most frequent food sources of *T. infestans*, associated with human rural dwellings [5]. At the end of each trial, arenas were cleaned with 10% bleach water, and once dry, followed with acetone to eliminate any remnant odors. A new shelter was placed for each trial.

### Data analysis

Analysis was focalized during the first hours of the scotophase, corresponding to peak activity [34] and oviposition moment [35], i.e. from 19:00 to 22:30 h, 210 minutes, totaling 35 h of video observation. Videos were viewed later and behavior was hand-recorded every 15 min (Additional file 1: Movie S1). Activity level was determined as the average number of individuals that left the shelter relative to the total of each subgroup during the first hours of the scotophase. At the end of each experiment, laid eggs were quantified.


**Additional file 1: Movie S1.** Fragment of the recorded video between 19:00 and 22:30 h of fasting females. Movement of unfed females with different reproductive states represented by different painted designs in their dorsal area. (MPG 3476 kb)


To assess the effects of treatment on activity levels of *T. infestans*, a generalized linear mixed-effects model [36] was fitted using function *lme* of package *nlme* [37] in R statistical software v. 3.4.3 (http://www.r-project.org). Average activity level for each night was included as the response variable, while “nutritional states” (fed and unfed females), “host” (absent or present), “reproductive state” (fertilized, virgin and fifth-stage nymph females), and their interactions, were included as fixed factors.

A random effect “trial” was added to account for the non-independence within group observations (three reproductive states, with and without host, were recorded from each trial). Non-significant interactions were removed, one at a time from higher to lower levels, to reduce the number of parameters to be estimated. Model assumptions, normality and homoscedasticity of residuals, were graphically checked. Due to residual variance increasing with increasing fitted value, a variance function “varExp” [37] was added to the model. Data were expressed as mean values ± standard error.

## Results and discussion

**Fig. 2:**
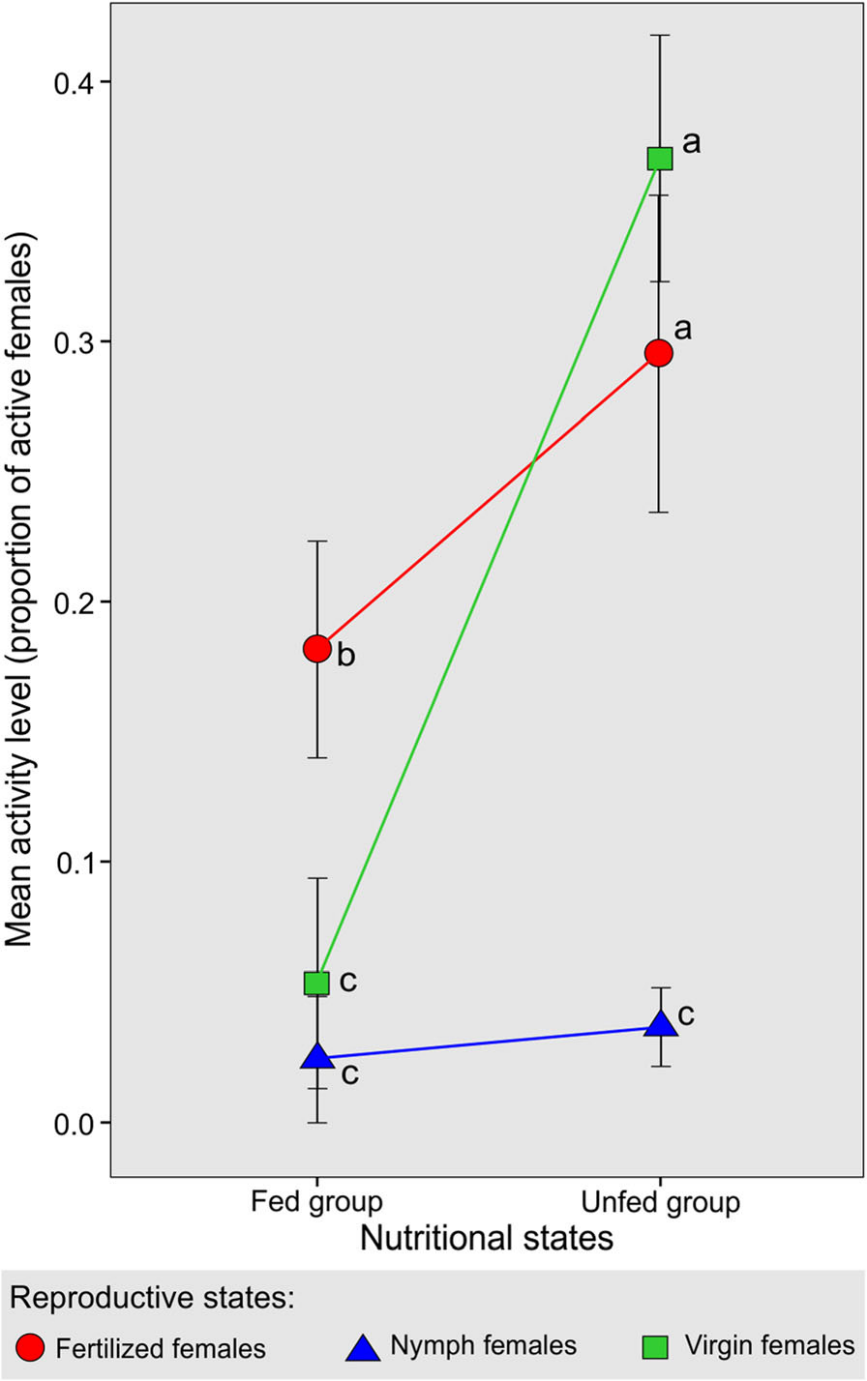
Activity levels in fed and unfed *Triatoma infestans* females according to their reproductive and nutritional states. Data are expressed as the mean ± standard error. Lines indicate interaction between nutritional and reproductive states. Different letters indicate significant differences between means *P* < 0.05

The effect of the nutritional state on females’ activity levels changed depending on their reproductive condition, although it was not possible to detect a difference between activity levels in the absence and presence of the host. The activity levels among the studied groups and subgroups were different. Figure 2 shows the significant interaction between reproductive and nutritional states (*F*_(2, 20)_ = 12.65, *P* < 0.01; Table 1). In fed *T. infestans*, fertilized females showed higher activity levels than virgin females and nymphs. Activity levels in unfed fertilized and virgin females did not differ significantly, although both were higher than those in nymphs. Comparing the fed and unfed groups, nymphs presented similar activity in both nutritional states, while virgin and fertilized unfed females presented higher activity levels with respect to their fed counterparts.

**Table 1:** Physiological factors for *Triatoma infestans* females’ activity levels and their significant interaction. Output of a general linear mixed effect model

Factor	*df*	*F*-value	*P*-value
(Intercept)	1, 20	60.59	<0.0001
Reproductive state	2, 20	37.41	<0.0001
Nutritional state	1, 3	14.24	0.0326
Host	1, 20	8.83	0.0076
Reproductive state*nutritional state	2, 20	12.65	0.0003

*Abbreviations*: df, degrees of freedom

Low activity of nymphs occurred in both nutritional states. Fifth-stage nymphs have a different physiological condition compared to adults. Nymphs show greater fasting resistance, 120 days *versus* 60 days in adults [38], allowing them to refrain from moving under potentially risky conditions such as in unknown environments. However, as reported by some authors, blood volume intake in nymphs is more than double of that in adults [3, 38], suggesting great voracity and a more active behavior in the search for food, a response that was not observed in the experimental context used here. The absence of a difference between nutritional groups could be due to an insufficient starving level. Lorenzo & Lazzari [31] reported an increased locomotor activity in nymphs exposed to a longer starvation level. Another cause of the low activity in nymphs could be the presence of adult females. In this context, adults in the same arena could have an inhibitory effect on the nymphs’ behavior. Intraspecific communication occurs in *T. infestans* through chemical and vibratory signals, mediating interactions between sexes, refuge localization and alarm signaling [29, 39–42]. Some authors reported that nymphs can respond to adults’ alarm pheromones (synthesized in Brindley’s glands), but do not produce them [29, 41]. In this context, we suggest that adults’ territorial signals could be affecting nymphs’ activity.

With respect to adults, fasting females showed higher activity levels than fed females. However, the difference in the activity levels between unfed and fed groups was much higher in the virgin females with fed virgins showing 8-fold lower activity than unfed ones (Fig. 2). The drive for movement in virgin females could be the need to find food and/or mate, although in our experimental conditions, fed virgins barely moved. This indicates that mate-searching motivation may not be the case, suggesting again a passive behavior in the reproductive encounter as other authors have reported [17–21]. Another possible explanation for the fed virgin female inactivity could be the fear of an unknown environment which could be interpreted as an increase in predation risk [42, 43]. It has been suggested that triatomines are night-feeders to avoid predation by hosts of diurnal habits such as hens [44–48].

In fertilized females, another motivational component could be involved, i.e. the need for oviposition. In these females, unfed insects showed greater activity than fed ones, although lower than in virgin females; unfed fertilized females presented 1.62-fold higher activity than fed ones (Fig. 2). It could be that fed fertilized females moved looking for oviposition sites (since the need for food is absent), and in unfed females feeding needs are added to the motivation for oviposition. It is known that oviposition exhibits a strong circadian rhythm with a peak at the same temporal window as dispersal and search for food [49]. Although other authors did not find a clear pattern of egg-laying distribution [28], in our study all eggs were found inside and around the shelter, suggesting a particular spatial egg-laying preference, strategically localized in the opposite corner to the host.

**Fig. 3:**
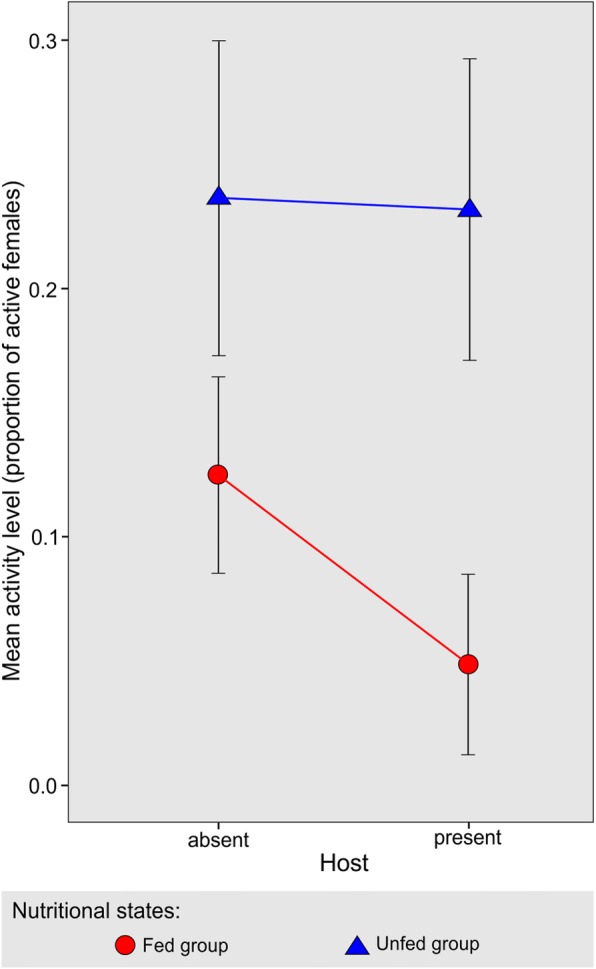
*Triatoma infestans* females’ activity levels in relation to host absence-presence according to their nutritional states. Data are expressed as mean ± standard error. Lines indicate repetitive measures on the same group of females when the host was either absent or present

Our study showed that in the host presence, *T. infestans* females reduced in 28% their mean activity levels compared to the host-absence situation (1.32 ± 0.21 *vs* 1.83 ± 0.23, respectively; *F*_(1, 20)_ = 8.83, *P* < 0.01). This effect was observed mainly in fed females, while the unfed females did not show any change in the host presence (Fig. 3), although these interactions were not significant (*F*_(1, 19)_ = 1.33, *P* = 0.26). Nuñez [27] reported similar activity levels in *R. prolixus* with equivalent fasting intervals; although as the activity recording continued, and consequently starvation intensified, activity was augmented, mainly in the host presence. Castillo-Neyra et al. [28] observed that host removal lead to increased *T. infestans* activity. However, in both mentioned studies [27, 28], methodological differences do not allow a direct comparison with the present data. For instance, we used fed control groups as well as different reproductive stages, host species and recording system.

In the foraging behavior of triatomines, two sequential phases could be identified. First, locomotion motivated by the need to localize potential food sources, represented in the absence of the host. Secondly, the actual detection of the host, observed in the second night when the hen was present. This detection could determine or not, a repulsion to the host, observed in fed and unfed females, respectively. Both groups were able to detect the host, although their behavioral expression was different depending on their nutritional status: unfed females maintained their activity levels while these were decreased in fed females. The former would continue their host search, while the latter would avoid it, possibly due to predation risk. Behavioral duality in triatomines, according to their nutritional status, has been shown in the attraction or repulsion to the carbon dioxide produced by hosts [49–51]. For the present data, increasing the number of replicates or the fasting interval would probably allow detection of significant interactions with the host factor.

Finally, we expected that *T. infestans* fertilized females would show a greater predisposition to move away in the presence of other females in different reproductive states as observed in a previous field dispersal study [7]. This relationship was fulfilled in fed fertilized females in controlled conditions. Due to the persistence of vector populations and their movement between habitats, intraspecific interactions studies on the activity levels as well as on other behavior variables (i.e. egg laying, response to host presence and to chemical and vibratory signals) are potentially useful to optimize vector detection and the control strategies.

## Conclusions

To our knowledge, the present data demonstrate for the first time that the reproductive condition of *T. infestans* females affects their activity levels depending on their nutritional state. Fasted females, both fertilized and virgin, showed higher activity levels than fed ones, while nymphs were mainly inactive. These data suggest that adults showed a higher predisposition to move in relation to nymphs. Virgin females were the most sensitive group, showing a dual behavior depending on their nutritional state. However, fertilized *T. infestans*, both fed and unfed, always showed activity. These laboratory results indicate that, as previously observed in the field, fertilized females may represent the highest epidemiological risk in the reinfestation of human dwellings.
